# Aneurysmal Subarachnoid Hemorrhage during the Shutdown for COVID-19

**DOI:** 10.3390/jcm11092555

**Published:** 2022-05-02

**Authors:** Erdem Güresir, Ingo Gräff, Matthias Seidel, Hartmut Bauer, Christoph Coch, Christian Diepenseifen, Christian Dohmen, Susanne Engels, Alexis Hadjiathanasiou, Ulrich Heister, Inge Heyer, Tim Lampmann, Sebastian Paus, Gabor Petzold, Dieter Pöhlau, Christian Putensen, Matthias Schneider, Patrick Schuss, Jochen Textor, Markus Velten, Johannes Wach, Thomas Welchowski, Hartmut Vatter

**Affiliations:** 1 Department of Neurosurgery, University Hospital Bonn, Venusberg-Campus 1, 53127 Bonn, Germany; alexis.hadjiathanasiou@ukbonn.de (A.H.); tim.lampmann@ukbonn.de (T.L.); matthias.schneider@ukbonn.de (M.S.); patrick.schuss@ukbonn.de (P.S.); johannes.wach@ukbonn.de (J.W.); hartmut.vatter@ukbonn.de (H.V.); 2 Emergency Department, University Hospital Bonn, Venusberg-Campus 1, 53127 Bonn, Germany; ingo.graeff@ukbonn.de (I.G.); matthias.seidel@ukbonn.de (M.S.); 3 Department of Neurology, Marien-Hospital Euskirchen, Gottfried-Disse Strasse 40, 53879 Euskirchen, Germany; hartmut.bauer@marien-hospital.com; 4 Clinical Study Core Unit, Study Center Bonn (SZB), University Hospital Bonn, Venusberg-Campus 1, 53127 Bonn, Germany; ccoch@gmx.net; 5 Emergency Medical Service Rhein-Sieg-Kreis, Kaiser-Wilhelm-Platz 1, 53721 Siegburg, Germany; c.diepenseifen@web.de; 6 Department of Neurology, LVR-Clinic Bonn, Kaiser-Karl-Ring 20, 53111 Bonn, Germany; christian.dohmen@lvr.de; 7 Department of Health City of Bonn, Berliner Platz 2, 53103 Bonn, Germany; dr.susanne.engels@bonn.de (S.E.); dr.inge.heyer@bonn.de (I.H.); 8 Emergency Medical Service City of Bonn, Berliner Platz 2, 53103 Bonn, Germany; u.heister@uni-bonn.de; 9 Department of Anesthesiology and Intensive Care Medicine, University Hospital Bonn, Venusberg-Campus 1, 53127 Bonn, Germany; christian.putensen@ukbonn.de (C.P.); markus.velten@ukbonn.de (M.V.); 10 Department of Neurology, St. Johannes-Hospital Troisdorf, Wilhelm-Busch-Strasse 9, 53844 Troisdorf, Germany; sebastian.paus@johannes-krankenhaus.com; 11 Department of Neurology, University Hospital Bonn, Venusberg-Campus 1, 53127 Bonn, Germany; gabor.petzold@ukbonn.de; 12 Department of Neurology, DRK-Kamillus-Clinic Asbach, Hospitalstraße 6, 53567 Asbach, Germany; dieter.poehlau@kamillus-klinik.de; 13 Department of Radiology, Gemeinschaftskrankenhaus Bonn, Prinz-Albert-Straße 40, 53113 Bonn, Germany; j.textor@gk-bonn.de; 14 Institute of Medical Biometry, Informatics and Epidemiology (IMBIE), University Hospital Bonn, Venusberg-Campus 1, 53127 Bonn, Germany; welchow@imbie.meb.uni-bonn.de

**Keywords:** aneurysmal subarachnoid hemorrhage, aneurysm, inflammation, neurosurgery, stroke, COVID-19, Poisson regression

## Abstract

The aim was to evaluate hospitalization rates for aneurysmal subarachnoid hemorrhage (SAH) within an interdisciplinary multicenter neurovascular network (NVN) during the shutdown for the COVID-19 pandemic along with its modifiable risk factors. In this multicenter study, admission rates for SAH were compared for the period of the shutdown for the COVID-19 pandemic in Germany (calendar weeks (cw) 12 to 16, 2020), the periods before (cw 6–11) and after the shutdown (cw 17–21 and 22–26, 2020), as well as with the corresponding cw in the years 2015–2019. Data on all-cause and pre-hospital mortality within the area of the NVN were retrieved from the Department of Health, and the responsible emergency medical services. Data on known triggers for systemic inflammation, e.g., respiratory viruses and air pollution, were analyzed. Hospitalizations for SAH decreased during the shutdown period to one-tenth within the multicenter NVN. There was a substantial decrease in acute respiratory illness rates, and of air pollution during the shutdown period. The implementation of public health measures, e.g., contact restrictions and increased personal hygiene during the shutdown, might positively influence modifiable risk factors, e.g., systemic inflammation, leading to a decrease in the incidence of SAH.

## 1. Introduction

The coronavirus disease 2019 (COVID-19) pandemic due to the novel coronavirus 2 (CoV2) caused a worldwide sudden and substantial increase in hospitalizations for pneumonia with and without multiorgan disease, which prompted the worldwide implementation of public health measures in order to reduce viral transmission. The German government response was a public shutdown, where several facilities including schools/day care, malls, and restaurants were closed, and people were encouraged to stay at home. This led to an overall reduction in emergency department visits, especially of patients with low acuity, as demonstrated among others by data from 29 university clinics and 7 large centers within Germany [1]. While this phenomenon served as an explanation for the decrease in admissions for acute myocardial infarction (AMI) during the COVID-19 pandemic, physicians were concerned about an increase in the case fatality and complication rates of AMI [2]. This led to the conclusion that countermeasures must be implemented in order to avoid further social impact.

Cardiovascular and cerebrovascular diseases such as aneurysmal subarachnoid hemorrhage (SAH) share the same modifiable risk factors, including arterial hypertension and smoking [3]. Increasing evidence points to inflammation as the leading factor in the development of intracranial aneurysms (IA), as well as in destabilization of the aneurysm wall leading to aneurysm rupture and SAH [4,5,6]. Systemic inflammation and inflammatory cascades promote endothelial dysfunction, plaque instability, as well as platelet and coagulation activity, leading to cardio- and cerebrovascular events [7]. Systemic inflammation in turn is known to be induced by smoking, infections (e.g., acute respiratory infections, influenza), and air pollution [6,8]. Therefore, guidelines for the treatment of AMI and cerebrovascular diseases recommend the reduction in modifiable risk factors, e.g., by cessation of smoking, and influenza vaccination in order to prevent the inflammatory trigger [9,10]. SAH is a devastating condition with high morbidity and mortality. The clinical hallmark of SAH is a history of severe headache with sudden onset. Half of the patients develop unresponsiveness for a period of time, and one third develop focal deficits, so that SAH patients were usually referred to hospital by emergency medical services [11,12,13]. Therefore, the rate of hospital admissions for SAH is not expected to vary due to the patients’ fear of COVID-19 transmission in the hospitals.

The aim of the present study was to analyze the effect of the COVID-19 pandemic shutdown on admission rates of SAH, as well as on its modifiable risk factors driving systemic inflammation as a trigger for aneurysmal instability and rupture.

## 2. Materials and Methods

**Figure 1:**
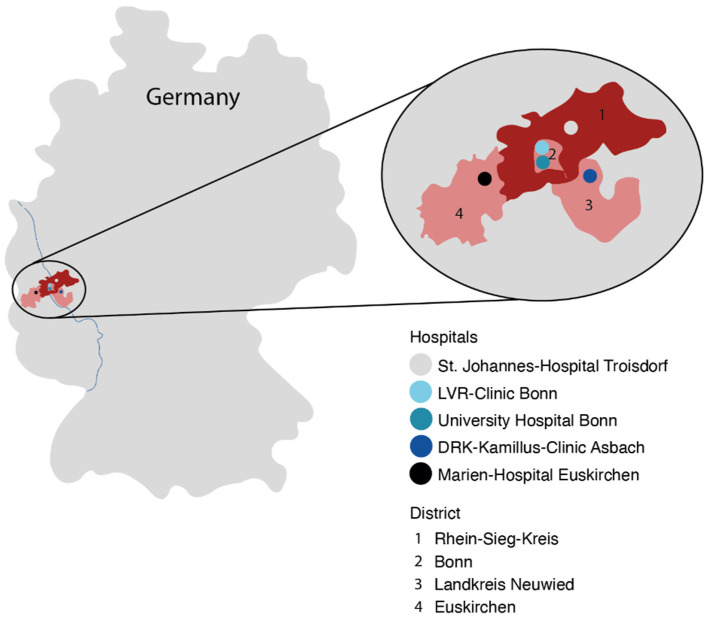
The neurovascular network “NeuroVask Bonn/Rheinland” (NVN) in Germany, consisting of neurology departments with acute stroke units, neuroradiology departments, cardiology departments, vascular surgery departments, and a neurosurgical department for neurosurgical intervention and treatment of patients with SAH in a metropolitan area with approximately 1.1 million inhabitants.

We performed a multicenter study within the neurovascular network “NeuroVask Bonn/Rheinland” (NVN) with a predefined catchment area. In order to improve access for neurovascular diseases (e.g., interventional recanalization therapies, endovascular and neurosurgical aneurysm treatment), the NVN was initiated by neurology departments with acute stroke units, neuroradiology departments, cardiology departments, vascular surgery departments, and a neurosurgical department for neurosurgical intervention and treatment of patients with SAH, in the metropolitan area Bonn/Rhein-Sieg with 1.1 million inhabitants (Figure 1). All patients with suspicious SAH were examined in the local centers of the NVN and triaged to the neuro emergency department (NED) of the University of Bonn when SAH was diagnosed or clarification in the primary hospital was not possible. Patients were stratified according to the Manchester Triage System (MTS) into five groups of urgency and dichotomized into “high urgency” (level red, orange, and yellow) and “low urgency” (level green and blue) [1]. All patients with diagnosed SAH within this network were categorized into “high urgency” and assigned to the neuro-intensive care unit (NICU) of the neurosurgical department of the university hospital Bonn via the NED.

SAH was diagnosed by computed tomography (CT) or lumbar puncture. All patients with spontaneous SAH underwent four-vessel digital subtraction angiography (DSA). Patients with traumatic origin of SAH were not included in this study.

Clinical data, including patient characteristics on admission and during treatment course, radiological features, and functional neurological outcome were prospectively collected and entered into a computerized database (IBM SPSS Statistics for Windows, Version 25.0. IBM Corp., Armonk, NY, USA). The World Federation of Neurological Surgeons (WFNS) scale was used in order to grade patients on admission. Consistent with the known incidence of SAH ranging between 4–10 per 100,000 patient years [14,15], the hospitalization rates for SAH within the catchment area of the NVN is around 7 per 100,000 patient years from 2013 to 2019, assessed within the prospectively conducted SAH database [5,16]. Baseline characteristics of SAH patients admitted in 2019 and 2020 are detailed in Table 1.

### 2.1. Time Periods

The public shutdown in Germany started in calendar week 12, 2020, with an impact on public life by extended health measures and social distancing in order to reduce viral transmission, and lasted until the end of calendar week 16. The calendar weeks 6 to 11, 2020, were defined as the weeks prior to the shutdown, but with increasing awareness including hygiene measures and social distancing.

The calendar weeks 17–21 and 22–26, 2020, were defined as the weeks after the public shutdown with relaxing shutdown measures.

### 2.2. Mortality

Data on mortality in the NVN area was derived from the Department of Health for comparable periods (1 February–30 April) in 2019 and 2020.

### 2.3. Preclinical Data

In the region of the NVN “NeuroVask Bonn/Rheinland”, patients with acute neurovascular events (e.g., stroke or subarachnoid hemorrhage) are managed by an emergency medical service (EMS) coordinated by synchronized and uniquely acting dispatch centers. Patients with neurovascular events are referred to the stroke units of the NVN nearby for primary diagnosis and treatment. In case of diagnosed SAH within the NVN, patients were referred to the neurosurgical department of the university of Bonn via NED.

Within the NVN, the transfer pathways for patients with potential neurovascular diseases are established, and an initial training was performed for the EMS in 2017. There was no change in the system configuration during the study period and the pandemic. In order to identify pre-hospital findings and mortality, as well as the assumed cause of death, the operational documents of the responsible EMS within the network were analyzed retrospectively within the corresponding periods of 2019 and 2020.

### 2.4. Influenza and Other Respiratory Viruses

Surveillance data on influenza, influenza-like-illness (ILI), and acute respiratory infections (ARI)—excluding SARS-CoV-2 infection—for the calendar weeks 6–26 were derived from the internet-based syndromic monitoring system “GrippeWeb” [17] of the Robert Koch Institute (RKI), the federal public health institute in Germany. The number of confirmed influenza reports submitted to the RKI were retrieved from Survstat (SurvStat@rki.de).

### 2.5. Air Pollution

Air quality data of the NVN area for the calendar weeks 6–26 2020 were derived from the State Agency for Nature, Environment and Consumer Protection of North Rhine Westphalia (LANUV). All measurements were based on hourly readings. The data included pollutants known to drive systemic inflammation: NO_x_, NO_2_, and particulate matter (PM10).

### 2.6. Statistics

Categorial variables are presented as absolute numbers and percentages. Continuous variables are presented as mean and standard deviation (SD) and compared by the Mann−Whitney U test. Hospitalization rate of SAH-patients was modeled by Poisson regression with log-link adjusted by time (year, weeks) using R software 4.0.4. The contrasts were estimated based on generalized linear hypothesis. IBM SPSS Statistics 25 was used for all other analyses.

### 2.7. Ethic

The present study was approved by the local ethic committee.

## 3. Results

Between 1 January 2018 and 17 May 2020, 164 patients with SAH were treated in the neurosurgical department of the University of Bonn/within the NVN.

### 3.1. SAH before Shutdown

**Figure 2:**
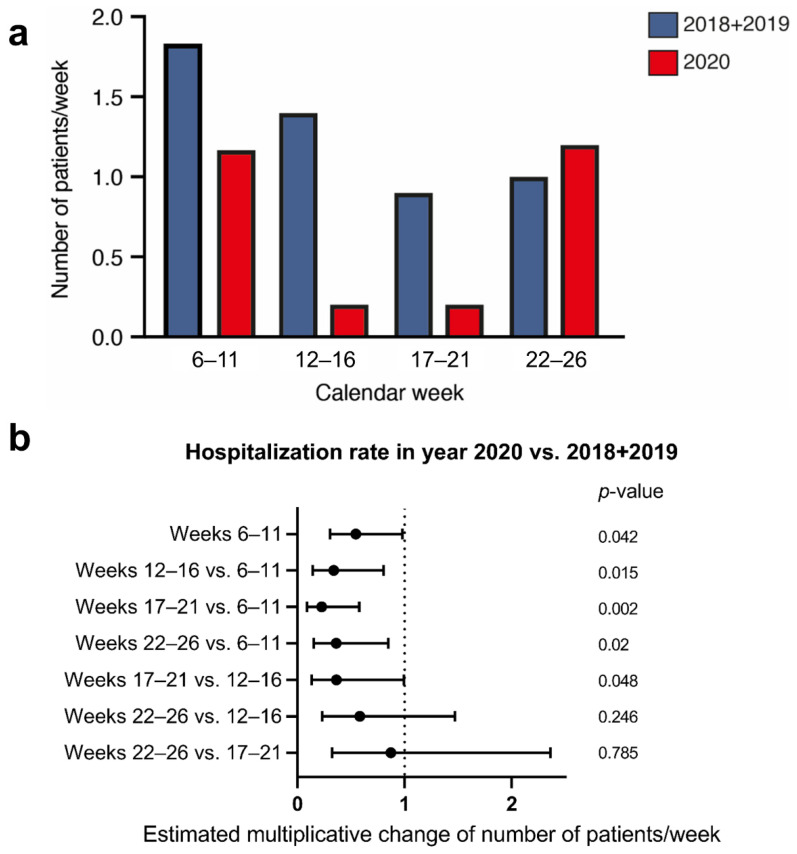
(**a**) Columns demonstrating hospitalization rates for aneurysmal subarachnoid hemorrhage (SAH) within the calendar weeks 6 to 26 in the years 2018 + 2019 (blue) and in the year 2020 (red). The public shutdown for the COVID-19 pandemic was announced for calendar weeks 12–16. The calendar weeks 6–11 were before, and the weeks 17–21 and 22–26 after, the public shutdown. (**b**) Forest plot visualizing the Poisson regression. The x-axis represents the multiplicative change of the hospitalization rate of SAH-patients at a given time period. Upper bounds of the confidence intervals below one correspond to *p*-values < 0.05.

There was no difference in hospitalization rates for SAH in the calendar weeks 6–11, 2020, before the shutdown (*n* = 7, 1.2 ± 0.7 patients/week) compared to the hospitalization rates in the corresponding calendar weeks of the years 2018 + 2019 (*n* = 22, 1.8 ± 1.3 patients/week) (Figure 2a).

### 3.2. SAH during the Shutdown

The hospitalization rate for SAH during the shutdown in the calendar weeks 12–16 and 17–21, 2020 (*p* = 0.015 and *p* = 0.002), was significantly lower compared to the corresponding calendar weeks in the years 2018 + 2019 according to the Poisson regression (Figure 2b).

### 3.3. Case Severity in the Neuro Emergency Department

The number of patients—MTS levels green and blue (low urgent)—admitted via the NED decreased in the corresponding calendar weeks of 2020, compared to 2019 (data not shown).

**Figure 3:**
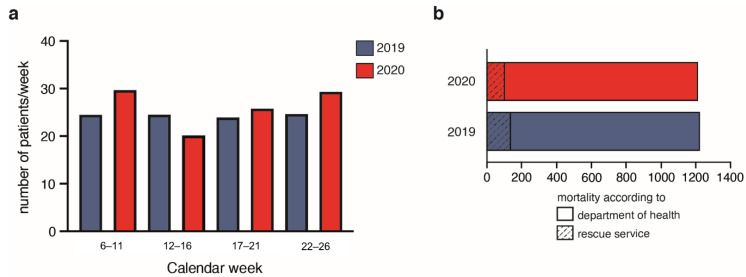
(**a**) Number of patients in the neuro emergency department (NED) admitted in the years 2019 (blue) and 2020 (red) with Manchester Triage Scale levels red, orange, and yellow (high urgent). (**b**) Mortality according to the Department of Health for the periods 1 February to April 30 2019 (blue) and 2020 (red). Out-of-hospital mortality is indicated as dashed lines within the columns.

The number of patients—MTS levels red, orange, and yellow (high urgent)—admitted via NED did not differ between the years 2019 and 2020 (Figure 3).

### 3.4. Mortality

According to the Department of Health, 1218 people died between 1 February 2019 and 30 April 2019 compared to 1205 between 1 February 2020 and 30 April 2020. There was no excess mortality within the area of the neurovascular network within these compared periods (Figure 3).

According to the data from the responsible rescue service and the fire department, out of hospital deaths were noted in 137 cases between 1 February 2019 and 30 April 2019, compared to 101 cases between 1 February 2020 and 30 April 2020 (Figure 3). According to the operational protocols of the emergency physicians, no patient died with the suspicion of subarachnoid hemorrhage (e.g., due to prior acute onset headache) in the time periods 1 February 2019 and 30 April 2019, as well as between 1 February 2020 and 30 April 2020.

### 3.5. Influenza and Other Respiratory Viruses

**Figure 4:**
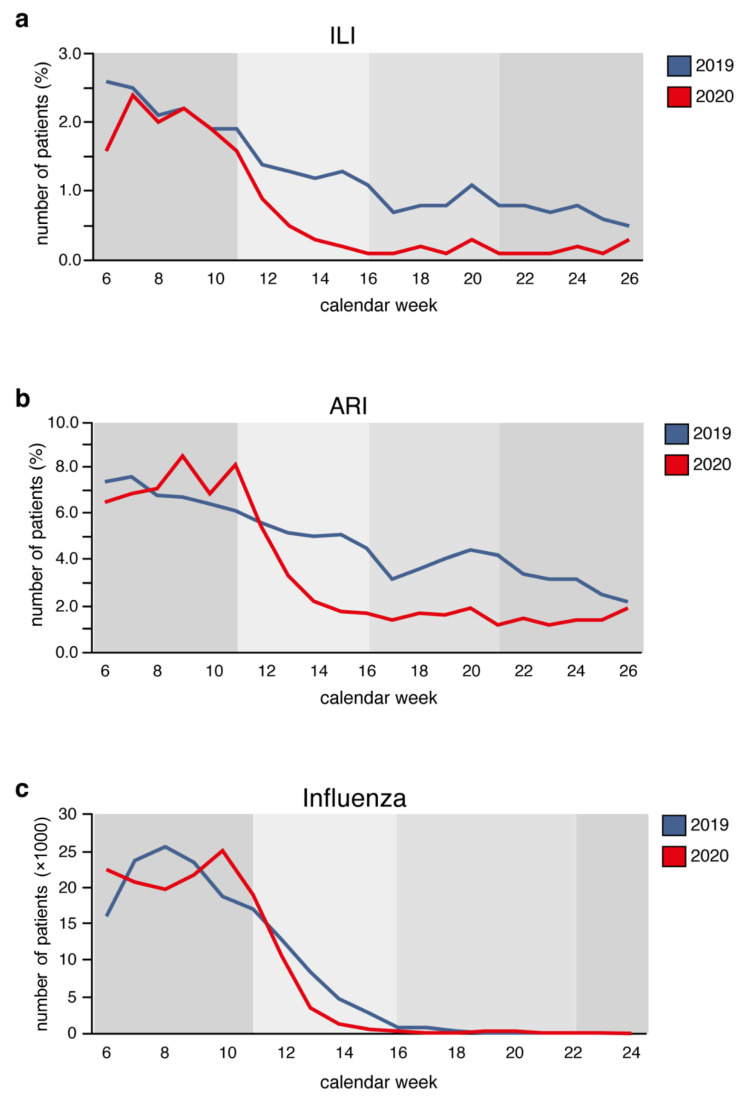
The rate of (**a**) influenza-like illness (ILI) and (**b**) acute respiratory illness (ARI) measured by GrippeWeb, excluding SARS-CoV-2 infections, as well as the number of (**c**) influenza reports submitted to the Robert Koch Institute within the weeks 6–26 in the years 2019 (blue) and 2020 (red). The public shutdown for the COVID-19 pandemic was announced for calendar weeks 12–16. The calendar weeks 6–11 were before, and the weeks 17–21 and 22–26 after, the public shutdown.

According to the internet-based syndromic monitoring system (Grippeweb), and data retrieved from Survstat, the reported rates of influenza, influenza-like-illness (ILI), and acute respiratory infections (ARI) have fallen substantially during the shutdown period compared to the year 2019 in Germany (Figure 4).

### 3.6. Air Pollution

**Figure 5:**
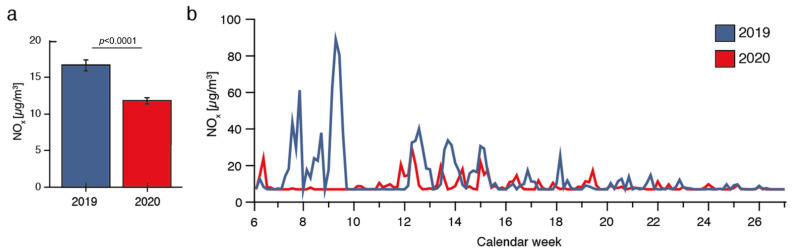
Air pollution within the calendar weeks 6–26 in the years 2019 (blue) and 2020 (red) during the public shutdown, exemplified for NO_x_ of the measuring station within the city of Bonn (data according to the State Agency for Nature, Environment and Consumer Protection of North Rhine Westphalia (LANUV)). Boxplots for mean values (**a**) and distribution of NO_x_ for the weeks 6–26 in the years 2019 (blue) and 2020 (red) (**b**).

According to the State Agency for Nature, Environment and Consumer Protection of North Rhine Westphalia (LANUV), air pollution, including NO_x_, NO_2_, and PM10 decreased during the shutdown period compared to the year 2019 and is exemplified for NO_x_ of the measuring station within the city of Bonn in Figure 5.

## 4. Discussion

The main finding of the present study is that admission rates for SAH decreased to one-tenth during the shutdown for the COVID-19 pandemic within a multicenter NVN in Germany.

While the proposed explanation leading to the reduced admission rates for SAH is speculative, it is evident that the public health measures in order to reduce viral transmission, i.e., public shutdown, contact restrictions, increasing awareness, and hygiene measures, reduced the risk factors for systemic inflammation, which in turn is a modifiable risk factor for cardio- and cerebrovascular diseases such as SAH.

There is growing evidence that SAH is the result of a chronic cerebrovascular inflammatory disease that leads to the development of aneurysms if other factors, e.g., arterial hypertension and genetic predisposition, among others, are present [6,18]. Aneurysms that go on to rupture might do so relatively early after formation when the aneurysm wall is weak and before healing processes begin, e.g., stimulated by an inflammatory trigger. Aneurysms that do not rupture at the initial stage, may reach a stable condition with a lower risk of rupture [4,6,19].

Therefore, the lower rates of hospital admissions for SAH might be the result of a lower rate of aneurysm formation, or of aneurysm rupture, both of which are thought to be the result of an inflammatory trigger.

Cardio- and cerebrovascular diseases share similar risk factors. Acute infections increase the risk of vascular events, i.e., AMI and Stroke [20]. For AMI, meta-analysis indicates the effectiveness of influenza vaccination up to 45%, being similar to smoking cessation [9,10]. For the prevention of cerebral infarction, vaccination for influenza is associated with lower risk of stroke events [21]. There is evidence that nearly all types of infectious agents increase the incidence of stroke [22]. ILI is associated with stroke events [23]. For SAH, one case-control study identified an association of recent respiratory infections and aneurysmal SAH [24]. Backes et al. [25] found an increased incidence of SAH during cold temperatures and epidemic influenza. The systemic inflammatory response induced by influenza accelerates endothelial injury by impairing vasodilatation by metabolic derangements, and enhances thrombotic tendencies through altered clotting factors and platelet dysfunction [26]. The substantial and early decreased rates of influenza, ILI, and ARI during the shutdown period in 2020 compared to the year 2019 might therefore be one part of an explanation for the decreased admission rates of SAH in the same period in 2020.

### 4.1. Neuro Emergency Department and Referral within the Neurovascular Network

Hospital admissions of patients—Manchester Triage Scale levels red, orange, and yellow (high urgent)—via NED did not differ between the years 2019 and 2020. Within the NVN, the transfer pathways for patients with potential neurovascular diseases are established, and an agreement between the hospitals exists for direct referrals and contemporaneous image transfer, if applicable. Within this area-wide network, the rescue services were urged to transfer patients between the treating centers, depending on the primary diagnosis. All patients with diagnosed SAH or the strong suspicion of SAH were referred to the neurosurgical center via NED. After retrospective review of the operational documents of the responsible EMS within the network, there was neither a difference in the referral of patients within the network, nor a higher pre-hospital mortality, which also promotes the assumption that the incidence of SAH decreased during the shutdown for the pandemic. This is also corroborated by the fact that there was no increase in overall mortality within the NVN area according to the Department of Health.

### 4.2. Air Pollution

Air pollution is a known environmental modulator of cardiac function [27] by systemic and pulmonary inflammation and oxidative stress, as well as a risk factor for cardiovascular morbidity and mortality [28,29]. Furthermore, evidence concerning the association of air pollution and brain pathology is growing [30]. Air pollutants (e.g., PM10, NO_x_, and others) were associated with increased hospitalization and adverse outcomes of stroke in a meta-analysis [31]. The pathophysiological mechanisms remain unclear; however, even short-term exposure to air pollution leads to an inflammatory response with increased levels of, e.g., C-reactive protein and IL-6 [32,33]. This systemic inflammation is held responsible for acute cardiovascular and cerebrovascular events such as ischemic stroke [31,34] and might also be a trigger for SAH [6,8].

Therefore, the reduced rates of admission for SAH might be explained by the environmental and behavioral changes during the pandemic; lower levels of air pollution and less physical and emotional strain at work may reduce the incidence of SAH [31,35].

Air pollution decreased markedly within the area of the NVN during the pandemic, as it was described for many countries world-wide [36,37]. Furthermore, the effect of wearing facial masks could also have provided protection from pollutants in addition to reduced air pollution by reduced traffic.

While there are no data on potential changes in tobacco use within the NVN, there are data in the literature indicating that the knowledge of COVID-19 is associated with a reduction in cigarette and e-cigarette use, as well as an increase in motivation to quit smoking [38]. In an online survey, researchers found that the desire to reduce the risk of SARS-CoV-2 infection prompted a quarter of respondents to reduce their cigarette consumption and more than one-third to increase their motivation to quit. It is unclear how successful and how stable this motivation for the reduction in smoking was or will be.

While one of the possible explanations for reduced rates of hospitalization for AMI might be the fear of SARS-CoV-2 infection, there is evidence that the reduced rates of hospitalization for SAH might be due to the reduction in modifiable risk factors driving systemic inflammation, which is a known risk factor for aneurysm rupture and SAH.

Patients with SAH might also be afraid of viral transmission, such as patients with AMI. However, in contrast to AMI, we did not observe delayed referral after SAH or excess mortality according to data from the Department of Health. Furthermore, there was no increase out of hospital mortality and no patient found dead with evidence of SAH in the time periods of 2019 and 2020 according to the data of the EMS.

Altogether, we provide data on reduced rates of hospitalization for SAH within a multicenter NVN during the COVID-19 pandemic. We hypothesize that the reduction in modifiable risk factors driving systemic inflammation (e.g., respiratory infections, air pollution, less physical strain at work) may play a key role in the reduction in SAH admissions by decreasing the risk of aneurysm rupture (see Graphical Abstract). Due to the similar risk factors for cardio- and cerebrovascular diseases, reduced systemic inflammation might also be a part of the explanation for reduced rates of hospital admissions during the COVID-19 pandemic for ischemic stroke and AMI.

The secondary increase in SAH admissions after the end of the shutdown to the same rates compared to the period before the pandemic might reflect the return of some of the modifiable risk factors for SAH. It is obvious that complex pathophysiological processes might not be turned on and off like a light switch, especially when SAH may result from a preexisting or a newly developed aneurysm that might differ in its risk factors or thresholds for rupture. Furthermore, we identified only a few of the known risk factors for SAH. We have no data on hypertension, smoking, and genetic predisposition of the population, as well of their stability or changes during the pandemic, within the NVN.

## 5. Conclusions

Hospitalization rates for SAH were reduced during the shutdown for the COVID-19 pandemic within a multicenter neurovascular network in Germany. The implementation of public health measures, e.g., contact restrictions and increased personal hygiene during the shutdown, might positively influence modifiable risk factors, e.g., systemic inflammation, leading to a decrease in the incidence of SAH.

## Figures and Tables

**Table 1:** Baseline characteristics of patients with aneurysmal subarachnoid hemorrhage.

	2019 Calendar Weeks 6–26	2020 Calendar Weeks 6–26
No. of patients	32	15
Mean age (years)	55 ± 12	56 ± 12
WFNS grade	3 ± 1	4 ± 2
Fisher grade 3	30 (94%)	14 (93%)
Female gender	18 (56%)	9 (60%)
Tobacco use	16 (50%)	7 (47%)
Arterial hypertension	16 (50%)	6 (40%)
Mean aneurysm size (mm)	6.7 ± 3.7	5.6 ± 0.5
Aneurysm location		
AComA + ACA	13 (41%)	7 (47%)
ICA	5 (16%)	3 (20%)
MCA	11 (34%)	3 (20%)
Posterior circulation	3 (9%)	2 (13%)
Time from ictus to hospitalization (h)	7 ± 2	5 ± 2

Values represent number of patients unless otherwise indicated. WFNS = World Federation of Neurological Surgeons; ACA = anterior cerebral artery; AcomA = anterior communicating artery; ICA = internal carotid artery; MCA = middle cerebral artery.

## Data Availability

The datasets generated during the current study are available from the corresponding author on reasonable request.
